# Characteristics of Ear, Nose, and Throat Comorbidities among Children with Allergic Rhinitis *

**DOI:** 10.1055/s-0045-1811517

**Published:** 2026-03-11

**Authors:** Siwanut Rattanaphibunsiri, Orathai Jirapongsananuruk, Kitirat Ungkanont, Archwin Tanphaichitr, Navarat Kasemsuk, Vannipa Vathanophas

**Affiliations:** 1 Department of Otorhinolaryngology, Faculty of Medicine Siriraj Hospital, Mahidol University, Bangkok, Thailand; 2 Department of Pediatrics, Faculty of Medicine Siriraj Hospital, Mahidol University, Bangkok, Thailand

**Keywords:** rhinitis, allergic, adenoid, tonsil, otitis media with effusion, rhinosinusitis

## Abstract

**Introduction:**

Children with allergic rhinitis (AR) may develop comorbidities due to chronic inflammation affecting other systems. Surveillance, early detection, and prompt treatment are essential in managing these conditions.

**Objectives:**

To investigate the prevalence and associated characteristics of ear, nose, and throat (ENT) disorders, particularly adenoid hypertrophy (AH), tonsillar hypertrophy (TH), otitis media with effusion (OME), and rhinosinusitis (RS) in children with AR. Also, to assess potential risk factors associated with these comorbidities.

**Methods:**

A total of 100 children aged 2 to 14 years with AR were enrolled. All patients underwent history taking, physical examination, and lateral skull X-ray processes by a pediatric otorhinolaryngologist.

**Results:**

There was a significantly higher incidence of TH in patients with moderate-to-severe persistent AR (54.5%) compared with those with mild intermittent AR (17.2%; *p* = 0.006). Furthermore, AH was observed in 51.7% of children with mild intermittent AR, significantly more than in other severity groups ( *p* = 0.037). The prevalence of TH, AH, OME, and RS was 41, 39, 8, and 1%, respectively. Additionally, OME was more common in suburban residents (14.3%) than in urban dwellers (2%; *p* = 0.029). The most common aeroallergen was the house dust mite *Dermatophagoides pteronyssinus* (89%).

**Conclusion:**

The most common ENT comorbidity in AR children is TH, being related substantially to the level of severity. The most prevalent aeroallergen was found to be house dust mite. The pattern of association between AR and ENT comorbidities highlights the essence of our research findings.

## Introduction

Allergic rhinitis (AR) is an inflammation of the nasal lining caused by an immunoglobulin E (IgE)-mediated immune response to allergens. It is classified as intermittent or persistent, according to the Allergic Rhinitis and its Impact on Asthma (ARIA) 2022 guidelines. Intermittent AR is defined as having symptoms for less than 4 days per week or for less than 4 consecutive weeks. The persistent type is defined as having symptoms for more than 4 days a week and for more than 4 weeks in a row. This condition has, so far, been classified as mild and moderate/severe, according to impairment on quality of life (QOL), sleep quality, and school or work performance. 1

Between 20 and 40 million people are affected by AR annually in the US, including 10 to 30% of adults and up to 40% of children. 2 The data from the phase I study of The International Study of Asthma and Allergies in Childhood (ISAAC) in 1991, when compared with the phase III study in 2001, demonstrated that there was an increasing prevalence of AR in our country children aged 6 to 7 years from 32.6 to 43.2% and 13 to 14 years from 43.4 to 57.4%. 3 The global prevalence of AR in the pediatric population has also increased significantly over the last 20 years. 4

Comorbidities may develop in children with AR as a result of the chronic inflammatory process in other related systems, including the pulmonary, auditory, and growth systems. 5 Asthma, chronic middle ear effusions, rhinosinusitis (RS), lymphoid hyperplasia, and obstructive sleep apnea are comorbidities, with behavioral and educational impact. 6 The importance of surveillance systems, early detection, and prompt treatment should be emphasized by identifying these associated comorbidities.

Previous researches in the United States and Europe have found a link between pediatric AR and a variety of ear, nose, and throat (ENT) comorbidities, including adenoid hypertrophy (AH), tonsillar hypertrophy (TH), otitis media with effusion (OME), and RS. 7 8 However, there have been limited researches on pediatric AR in Asia, where the craniofacial anatomy and environment differ. 9 The data should help pediatricians and otorhinolaryngologists identify subsequent comorbidities. Early detection, treatment, and long-term monitoring of these ENT comorbidities in children with AR may reduce the associated undesirable complications.

The presenr study aimed to investigate the prevalence and associated characteristics of ENT disorders, including TH, AH, OME, and RS in children with AR. Furthermore, common aeroallergen sensitization was identified in children with AR, and the correlations between ENT comorbidities and particular aeroallergen sensitization, household smoking, living location, family history of atopic disorders, and AR severity were assessed.

## Methods

This cross-sectional study was conducted in the pediatric allergy clinic at the Department of Pediatrics of a tertiary care hospital, from August 2019 to 2020. The study was approved by Siriraj Institutional Review Board, under the COA no. Si 607/2019. Also, written consent forms were obtained from all participants before enrollment.

### Eligibility Criteria

Children aged 2 to 14 years with a clinical diagnosis of AR defined by relevant positive signs and symptoms including nasal obstruction, rhinorrhea, sneezing, and itching, accompanied by a positive skin prick test (SPT), were included in the study. However, those who were excluded entailed participants with craniofacial anomalies, Down's syndrome, and a history of previous ENT surgeries including adenoidectomy, tonsillectomy, myringotomy, and sinonasal surgery.

### Sample Size Calculation

The sample size was calculated by using data from previous studies by Modrzynski et al., 7 Tomonaga et al., 10 and Pherwani et al., 11 with a proportion of AH: 0.70, OME: 0.20, and RS: 0.23, respectively. The sample size calculation was performed using the nQuery Advisor (Statsols) software. A single proportion model was applied for each condition to determine the largest required sample size with a 95% confidence level and a 10% margin of error. Based on these calculations, the maximum required sample size was determined to be 88, and we enrolled 100 participants to account for potential data loss and to enhance precision.

### Methods

All patients underwent a medical history analysis, physical examination, and lateral skull X-ray. This last procedure was standardized to ensure consistency in measurement. All radiographs were performed using a general X-ray unit operated by well-trained radiologic technicians, following a consistent protocol for patient positioning and exposure parameters. The images were then interpreted using the Fujioka method to calculate the adenoid-nasopharyngeal (A/N) ratio, ensuring uniformity in assessment across all participants.

All relevant information was obtained through patient interview and physical examination conducted by the investigators on the day of enrollment, including age, sex, age of diagnosis, symptoms, severity of AR, family history of atopic diseases, hometown, underlying illnesses, weight, height, and aggravating factors. No retrospective chart review was used to ensur consistency and accuracy in data collection.

The aeroallergen sensitization pattern was assessed using the skin prick test (SPT) or serum specific IgE in all participants. The allergen panel included *Dermatophagoides pteronyssinus* (DP), *Dermatophagoides farinae* (DF), American cockroach, German cockroach, cat, dog, and mold. A positive SPT was defined as a wheal diameter at least 3 mm greater than the negative control.

The prevalence of TH, AH, OME, and RS were recorded, and specific investigations were performed for each condition by a pediatric otorhinolaryngologist to find out the pattern of association between the ENT disorders and AR children.

#### Diagnostic Definitions

- The tonsillar size was evaluated using the Brodsky grade scale. 12 Hypertrophy was defined by tonsillar grades 3+ and 4 + .
- The A/N ratio was calculated from lateral skull X-ray using the Fujioka method. 13 Results over 0.7 were considered AH.
- Patient's ears were inspected with a pneumatic otoscope. An abnormal otoscopic exam was defined as a cloudy tympanic membrane (TM) with fluid levels or bubbling and a diminished or absent movement of the TM in response to pressure change. 14
- We defined RS by signs and symptoms, according to the European Position Paper on Rhinosinusitis and Nasal Polyp (EPOS) 2020 guideline. 15

All physical examinations, including the grading of TH and assessment for OME, were performed by a pediatric otorhinolaryngologist using standardized and widely accepted clinical criteria. Tonsillar size was graded using the Brodsky scale, while OME was assessed with pneumatic otoscopy. The examiners were not blind to the patients' clinical background. However, the assessments were based on objective criteria routinely used in clinical practice and considered reproducible.

### Statistical Analysis

All statistical analyzes were performed with the Statistical Package Social Sciences (SPSS, IBM Corp.) software, version 26. A *p* -value of less than 0.05 was considered statistically significant. A descriptive analysis of continuous variables was performed, being demonstrated as mean and standard deviations (SDs), or medians and ranges, as appropriate. Categorical variables were reported as frequencies and percentages. The association between ENT comorbidities and specific aeroallergens, smoking at home, hometown, and family history of atopic diseases was evaluated using Fisher's exact test. The linear-by-linear association chi-squared test was used to assess the association between the severity of AR and ENT comorbidities.

## Results

**Fig. 1:**
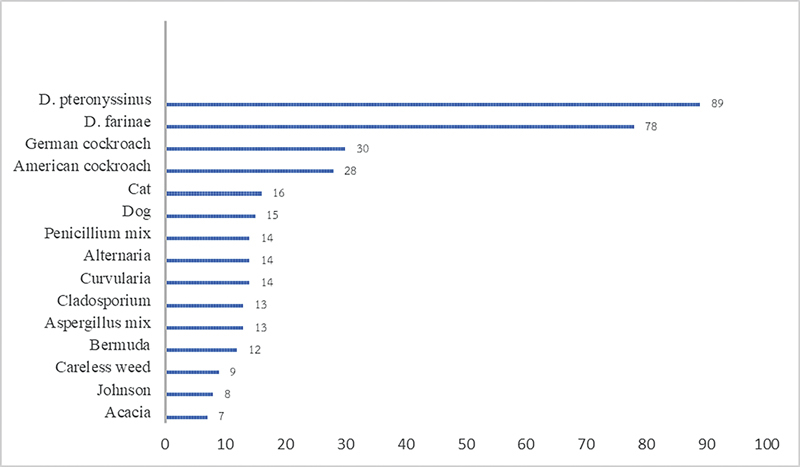
Sensitized aeroallergens in children with allergic rhinitis.

There were 100 children with AR included. The average age was 8.7 ± 2.7 years, and 69% of were boys. All patients had positive SPT results and were treated by pediatric allergists. Baseline characteristics and demographic data are shown in Table 1 . The prevalence of TH, AH, OME, and RS in pediatric patients with AR was 41, 39, 8, and 1%, respectively. The aeroallergen sensitization pattern is shown in Fig. 1 . The most common sensitized aeroallergen in children with AR was 89% DP, followed by 78% DF

**Table 1:** Patient demographic and clinical characteristics ( *N* = 100)

Age, mean ± SD, years	8.7 ± 2.7
**Sex, N (%)**	
Male	69 (69.0)
Female	31 (31.0)
** Weight status category, ^a^ N (%) **	
Normal	73 (73.0)
Overweight	20 (20.0)
Obesity	7 (7.0)
**Underlying disease, N (%)**	74 (74.0)
Asthma	44 (59.5)
Food allergy	12 (16.2)
AD	10 (13.5)
ADHD	10 (13.3)
Others	25 (33.8)
**Family history of atopic disease, N (%)**	66 (66.0)
AR	59 (89.4)
Asthma	17 (25.8)
AD	4 (6.1)
**Home smoker, N (%)**	31 (31.0)
**Hometown, N (%)**	
City	51 (51.0)
Others	49 (49.0)
**Classification of AR, N (%)**	
Mild intermittent	60 (60.0)
Mild persistent	22 (22.0)
Moderate-severe intermittent	10 (10.0)
Moderate-serve persistent	8 (8.0)

**Abbreviations:** AD, atopic dermatitis; ADHD, attention deficit hyperactivity disorder; AR, allergic rhinitis; SD, standard deviation.

**Note:**^a^ Weight status was categorized based on % weight for height.

The findings revealed that TH has become more common as the severity of AR increased ( *p* = 0.006). On the other hand, AH was obviously observed in half of the children with mild intermittent AR ( *p* = 0.037). The association between OME and AR children had no statistical significance. Furthermore, due to the marginal finding of RS in AR children, the association was so insignificant that the result cannot be clarified. Finally, the association between AR severity and ENT comorbidities is shown in Table 2 .

**Table 2:** Association between classification of AR and ENT disorders

Classification of AR	TH ^a^ N (%)	AH ^a^ N (%)	OME ^a^ N (%)
Mild intermittent	19 (31.7)	30 (50.0)	4 (6.7)
Mild persistent	10 (45.5)	4 (18.2)	1 (4.5)
Moderate-severe intermittent	6 (60.0)	3 (30.0)	1 (10.0)
Moderate-severe persistent	6 (75.0)	2 (25.0)	2 (25.0)
***p*** **-value**	0.006*	0.037*	0.151

**Abbreviations:** AH, adenoid hypertrophy; AR, allergic rhinitis; ENT, ear, nose, and throat; OME, otitis media with effusion; TH, tonsillar hypertrophy.

**Note:** Analyzed by ^a^ Linear-by-Linear association chi-squared test.

The association between ENT comorbidities and particular aeroallergens (DP and DF), household smoking, hometown location, and family history of atopic disorders was also assessed. There was no statistically significant association between ENT comorbidities and almost all of these factors, except for the association between OME and hometown locations. Approximately 2% of the children living in cities experienced OME, compared with almost 14.3% of those living outside them ( *p* = 0.029). Detailed data are provided in Table 3 .

**Table 3:** Association between OME and specific aeroallergens (DP and DF), home smoker, hometown, and family history of atopic disease

Comorbidities	DP *N* = 89	DF *N* = 78	Home smoker *N* = 31	City *N* = 51	Family history of atopic diseases *N* = 66
**OME**	7 (7.9)	8 (10.3)	2 (6.5)	1 (2.0)	6 (9.1)
***p*** **-value**	1.000	0.194	1.000	0.029*	0.713

**Abbreviations:** AH, adenoid hypertrophy; AR, allergic rhinitis; DF, *Dermatophagoides farinae* ; DP, *Dermatophagoides pteronyssinus* ; ENT, ear, nose, and throat; OME, otitis media with effusion; TH, tonsillar hypertrophy.

**Notes:** Analyzed by Fisher's exact test. * *p* -values < 0.05 were considered statistically significant.

## Discussion

As one of the most common chronic diseases in children, the global prevalence of AR has increased significantly in the last 20 years. 3 Comorbidities may also unavoidably occur consequently. There are various manifestations in our otorhinolaryngological practice. Therefore, early diagnosis, treatment, and follow-up are critical and should be performed in a multidisciplinary team to minimize possible additional complications that can affect the quality of life of children and their parents.

### Allergic Rhinitis and Adenotonsillar Hypertrophy

As the palatine tonsils and nasopharyngeal tonsil (adenoid) constitute the major part of Waldeyer's ring or nasal-associated lymphoid tissue (NALT), they contribute to the early development of immunity against inhaled microorganisms. 16 Upper airway lymphoid hypertrophy with prominence of adenoidal and tonsillar tissue is common in AR children. 6 Nasal blockage, rhinolalia clausa, open-mouth breathing, snoring, and adenoid facies are the symptoms commonly associated with adenotonsillar hypertrophy (ATH). 16 The most common cause of pediatric obstructive sleep apnea (OSA) is ATH which, when untreated, can cause neurocognitive impairments, behavioral problems, cardiovascular disease, and decreased quality of life in the long-term. 17

A previous study by Lack et al. demonstrated that almost 30% of patients with TH had AR, compared with only 8% of those without it. 6 In contrast, Ameli et al. showed that tonsillar enlargement was not associated with AR. 18 Until now, the relationship between AR and TH has been controversial, with limited data on the proportion in pediatrics. The study showed that the prevalence of TH in AR patients is 41%, the highest one among ENT comorbidities. Additionally, the association of TH in AR children increased along with its severity.

However, it contrasted with AH in AR children in that the association increased in mild AR. Modrzynski et al. discovered a considerable relationship between AH and AR, with a 32.9% rate in children. 7 This finding is comparable to the present study's result, which demonstrates that AH was present in 39% of children with AR.

### Allergic Rhinitis and Otitis Media with Effusion

The most common cause of acquired hearing loss in children is OME. However, the etiology remains multifactorial and controversial. 19 The main cause of OME is believed to be Eustachian tube dysfunction. Upper respiratory tract infections (URTI), mechanical obstruction of the nasopharynx due to AH or craniofacial deformities such as cleft lip and palate and Down syndrome, allergies, immunologic deficiencies, bacterial biofilms, and genetic factors are also risk factors. 20 According to the concept of global airway allergy, it can be expected that inflammation will occur in the middle ear. 16

A previous study from Japan found OME in up to 21% of children with AR. On the other hand, 50% of children who were diagnosed with OME were found to have AR. 10 Another study mentioned that the prevalence of OME and allergy in children can range from 5 to 80%, with the average being around 23%. 5 Approximately 2% of our participants experienced OME, which was in line with previous research findings. Because all the participants in this study had taken medication before being enrolled, it may improve nasal obstruction and AH, which are its cause.

### Allergic Rhinitis and Rhinosinusitis

Allergic inflammation of the nasal mucosa may cause congestion, resulting in decreased mucus outflow at the osteomeatal complex. 5 Caruso et al. showed that AR was present in 56% of patients with chronic RS (CRS). 8 This in children with chronic and recurrent RS was significantly higher than in those with the acute and subacute types. 21 Compared with the general population, whose CRS was discovered up to 6%, those with AR appeared to have sinus problems more frequently. 16

The study found that only 1% of children with AR had RS, compared with 23% in a report by Pherwani et al. 11 This could be due to some participants being partially treated by pediatric allergists prior to enrollment, as well as the challenge of detecting AR and RS due to overlapping symptoms. House dust mites were the most common allergens, which is consistent with recent research in Asia and around the world. 22 The researchers tried to observe the association between AR classification and TH, which became significantly more common as the severity of AR increased. It was noted that AH could be found in half of the children with mild intermittent AR. This may be in part due to intranasal corticosteroids (INCS) and leukotriene receptor antagonists (LTRA) being used to reduce the size of the adenoid. 23 As a result, it was hypothesized that children in the mild-intermittent group would use fewer INCS or receive no LTRA due to their less severe symptoms. Eventually, the AH cases were found less often than they should have been. However, the TH cases were found conversely, according to the assumption that both kinds of drugs do not affect it.

The present study assessed the prevalence and the associated characteristics of ENT comorbidities, especially TH, AH, OME, and RS, in children with AR. The prevalence of TH was as high as 41%, becoming significantly more common as the severity of AR increased ( *p* = 0.006). This may be because AH children were usually prescribed nasal steroid and related mediation, which do not alleviate TH. Therefore, AH was observed in half of the children with mild intermittent AR ( *p* = 0.037). The most common aeroallergen was the house dust mite (HDM). Furthermore, there was a statistically significant association between OME and place of residence.

In our study, all children with AR underwent lateral skull X-ray as part of a standardized assessment protocol. This approach was based on the rationale that previous literature has reported a high prevalence of AH in pediatric patients with AR, even in the absence of overt clinical symptoms, such as mouth breathing or OME.

We aimed to comprehensively evaluate subclinical cases that may otherwise be missed through symptom-based screening alone. We acknowledge the ethical considerations of exposing children to radiation. However, the lateral skull X-ray delivers a relatively low dose, and the potential benefit of early detection of AH outweighed this minimal risk. Moreover, imaging was performed using standard pediatric radiographic protocols by trained technicians to ensure safety and consistency.

The differences in findings compared with previous literature may be attributable to variations in assessment methods, leading to the higher prevalence of AH observed among children with mild intermittent AR in our study. Furthermore, AH was assessed using lateral skull X-ray and calculated via the A/N ratio, which may differ in sensitivity and specificity from the endoscopic or clinical examinations used in other studies. Additionally, the use of medications such as intranasal corticosteroids and leukotriene receptor antagonists prior to enrollment may have influenced adenoid size and contributed to this observation. These methodological differences should be considered when comparing studies.

This study has several limitations. First, these are data from a single tertiary institution, which may affect the generalizability of the result. Second, there was no comparison group in the one used as a control. Third, the results of physical examinations may be influenced by the various assessors. Future research that includes a control group at other levels of health care is suggested. Finally, we anticipate that identifying these linked comorbidities will highlight the importance of surveillance, early detection, and prompt treatment.

## Conclusion

The most frequent comorbidity of ENT in children with AR is TH, which was also significantly associated with the severity of AR and can entail the pattern of association of its classification. The most prevalent aeroallergen was HDM. A measurement of the association between AR and ENT comorbidities could highlight the significance of our results. Future research involving a control group at other levels of health care is suggested.
